# Can I solve my structure by SAD phasing? Anomalous signal in SAD phasing

**DOI:** 10.1107/S2059798315019269

**Published:** 2016-03-01

**Authors:** Thomas C. Terwilliger, Gábor Bunkóczi, Li-Wei Hung, Peter H. Zwart, Janet L. Smith, David L. Akey, Paul D. Adams

**Affiliations:** a Bioscience Division, Los Alamos National Laboratory, Mail Stop M888, Los Alamos, NM 87545, USA; b Department of Haematology, University of Cambridge, Cambridge Institute for Medical Research, Wellcome Trust/MRC Building, Cambridge CB2 0XY, England; c Physics Division, Los Alamos National Laboratory, Mail Stop D454, Los Alamos, NM 87545, USA; d Physical Biosciences Division, Lawrence Berkeley National Laboratory, Berkeley, CA 94720, USA; e Life Sciences Institute, University of Michigan, Ann Arbor, MI 48109, USA; f Department of Biological Chemistry, University of Michigan, Ann Arbor, MI 48109, USA

**Keywords:** SAD phasing, anomalous signal, anomalous phasing, solving structures

## Abstract

A key challenge in the SAD phasing method is solving a structure when the anomalous signal-to-noise ratio is low. A simple theoretical framework for describing measurements of anomalous differences and the resulting useful anomalous correlation and anomalous signal in a SAD experiment is presented. Here, the useful anomalous correlation is defined as the correlation of anomalous differences with ideal anomalous differences from the anomalous substructure. The useful anomalous correlation reflects the accuracy of the data and the absence of minor sites. The useful anomalous correlation also reflects the information available for estimating crystallographic phases once the substructure has been determined. In contrast, the anomalous signal (the peak height in a model-phased anomalous difference Fourier at the coordinates of atoms in the anomalous substructure) reflects the information available about each site in the substructure and is related to the ability to find the substructure. A theoretical analysis shows that the expected value of the anomalous signal is the product of the useful anomalous correlation, the square root of the ratio of the number of unique reflections in the data set to the number of sites in the substructure, and a function that decreases with increasing values of the atomic displacement factor for the atoms in the substructure. This means that the ability to find the substructure in a SAD experiment is increased by high data quality and by a high ratio of reflections to sites in the substructure, and is decreased by high atomic displacement factors for the substructure.

## Abstract 2

The useful anomalous correlation and the anomalous signal in a SAD experiment are metrics describing the accuracy of the data and the total information content in a SAD data set and are shown to be related to the probability of solving the anomalous substructure and the quality of the initial phases.

## 1. Introduction   

### 1.1. Single-wavelength anomalous diffraction   

The single-wavelength anomalous diffraction (SAD) method is a remarkably powerful approach to macromolecular structure determination (Hendrickson & Teeter, 1981 ▸; Wang, 1985 ▸; reviewed in Dauter *et al.*, 2002 ▸; Hendrickson, 2014 ▸). It has become the dominant method for the determination by X-ray diffraction of structures that are not closely related to any structure already present in the Protein Data Bank (PDB; Berman *et al.*, 2000 ▸), accounting for 73% of such new structures by 2013. In the SAD approach, the X-ray wavelength is tuned to be at or near an absorption edge of an element that is present at a limited number of sites in the macromolecule. Depending on the element and the wavelength, part of the scattering from the atoms in this substructure will then be shifted in phase by π/2 relative to the ‘normal’ scattering from other atoms in the structure. This results in a difference in intensities for reflections related by inversion which otherwise would have identical intensities (Bijvoet, 1954 ▸). The anomalous differences, which correspond to the atoms in the sub­structure (Kartha & Parthasarathy, 1965 ▸; North, 1965 ▸; Strahs & Kraut, 1968 ▸), are then used to find the locations of these substructure atoms (Weeks *et al.*, 1993 ▸; Terwilliger & Berendzen, 1999 ▸; Schneider & Sheldrick, 2002 ▸; Grosse-Kunstleve & Adams, 2003 ▸). The substructure and the anomalous differences are then used together to estimate crystallographic phases for the entire structure (Hendrickson & Teeter, 1981 ▸; Wang, 1985 ▸; Otwinowski, 1991 ▸; de La Fortelle & Bricogne, 1997 ▸; Furey & Swaminathan, 1997 ▸; McCoy *et al.*, 2004 ▸; Pannu & Read, 2004 ▸). An electron-density map can then be calculated with these phases and the averages of structure factors for Bijvoet pairs. This electron-density map is normally then improved by density-modification techniques (Wang, 1985 ▸; Cowtan & Main, 1996 ▸; Abrahams & Leslie, 1996 ▸; Terwilliger, 2000 ▸; Cowtan, 2010 ▸) and interpreted in terms of an atomic model (see, for example, Jones *et al.*, 1991 ▸; Perrakis *et al.*, 1999 ▸; Emsley *et al.*, 2010 ▸; Terwilliger *et al.*, 2008 ▸; Cowtan, 2006 ▸; Langer *et al.*, 2008 ▸).

There are several crucial steps in carrying out a SAD experiment (Dauter *et al.*, 2002 ▸; Liu *et al.*, 2011 ▸; Hendrickson, 2014 ▸). Some of these are experimental steps, such as having a crystal with well ordered anomalous scatterers and making accurate measurements of the intensities of Bijvoet pairs of reflections without substantial radiation damage (see, for example, Debreczeni *et al.*, 2003 ▸; González, 2003 ▸; Garman, 2003 ▸; Krojer *et al.*, 2013 ▸). Subsequent crucial steps involve analysis of the experimental data, decisions about which data to include, finding the locations of the atoms in the substructure, choosing the correct hand of the substructure and obtaining sufficiently accurate phase information to allow density-modification procedures to improve the phases and yield an interpretable electron-density map (Hendrickson, 2014 ▸). Here, we describe a formulation for the useful anomalous signal and test it with several solved structures. In the accompanying manuscript (Terwilliger *et al.*, 2016 ▸), the application of the formulation to data sets for unsolved structures is described.

### 1.2. Measures of signal and noise in anomalous data   

The anomalous differences between Bijvoet pairs of reflections are generally small (typically 1–5%), so a key overall consideration in a SAD experiment is obtaining sufficient signal. Additionally, anomalous differences, even if perfectly measured, are not the same as the structure factors for the anomalously scattering substructure, so these differences can be thought of as having a high level of intrinsic noise. Although these factors have been recognized for some time, it has not been fully clear which metrics best describe the signal in a SAD experiment and which values of these metrics indicate that the substructure will be solved and a sufficiently accurate electron-density map obtained.

#### 1.2.1. Metrics predictable from experimental setup   

A measure of signal in anomalous data that can be estimated in advance of carrying out an experiment is the Bijvoet ratio 〈|*F* ^+^ − *F* ^−^|〉/〈*F*〉 (Hendrickson & Teeter, 1981 ▸; Wang, 1985 ▸). As noted by Zwart (2005 ▸) and Dauter (2006 ▸), this measure is useful for obtaining a general idea of how large the anomalous signal might be, but the experiment and errors in measurement substantially affect the actual anomalous signal. Additionally, this ratio is strongly affected by the atomic displacement factors of atoms in the anomalous substructure (Shen *et al.*, 2003 ▸; Zwart, 2005 ▸).

#### 1.2.2. Metrics calculated from measured anomalous differences   

An important set of metrics for signal in anomalous data are based on estimates of the accuracy of measured anomalous differences. One of these is the ratio of the mean anomalous difference to the mean estimated uncertainty in the difference 〈|Δ_ano_|〉/〈σ_ano_〉 (Schneider & Sheldrick, 2002 ▸; Wang, 1985 ▸). This can be used to identify the resolution to which the anomalous differences are useful (Schneider & Sheldrick, 2002 ▸). A related measure is based on a normal probability plot of normalized anomalous differences (Howell & Smith, 1992 ▸). Another related metric used to identify which anomalous differences are useful is the ‘measurability’ of an anomalous data set (Parthasarathy & Parthasarathi, 1974 ▸; Zwart, 2005 ▸), which describes the fraction of anomalous differences that are very accurately measured (those with anomalous differences at least three times the magnitude of their uncertainties). A different metric used to identify whether anomalous differences are useful is a comparison of the merging χ^2^ considering Bijvoet pairs as being equivalent with the χ^2^ considering them separately (Otwinowski & Minor, 1997 ▸).

An estimate of signal that is based on experimental measurements but that does not require the use of experimental estimates of uncertainty is the anomalous scattering ratio, *R*_as_ (Fu *et al.*, 2004 ▸). This measure is the ratio of differences among measurements of equivalent acentric reflections compared with measurements of equivalent centric reflections. As centric reflections have no anomalous differences but the errors in measurement are likely to be similar to those of the acentric reflections, this comparison can be a good indicator of the signal in the anomalous data. Its value for a series of data sets for zinc-free insulin crystals (Fu *et al.*, 2004 ▸) was closely related to whether the substructure could be determined using *SHELXD* (Schneider & Sheldrick, 2002 ▸).

A very powerful measure of signal in an anomalous data set that also does not require estimates of experimental uncertainties is the correlation of anomalous differences measured at different wavelengths, obtained from different crystals, measured in different regions of reciprocal space, or simply measured more than once. The correlation of anomalous differences between data collected in different X-ray diffraction images obtained from crystals derivatized with heavy atoms was used some time ago to confirm the presence of anomalous differences using data collected on film (Colman *et al.*, 1972 ▸; Buehner *et al.*, 1974 ▸). The correlation of anomalous differences measured at different X-ray wavelengths of a MAD experiment were used in *SOLVE* (Terwilliger & Berendzen, 1999 ▸) and *SHELXD* (Schneider & Sheldrick, 2002 ▸) to identify the resolution to which the anomalous differences were likely to be useful (Dauter, 2006 ▸). More recently, the half-data-set anomalous correlation (Evans, 2006 ▸), obtained by dividing an unmerged data set into two parts and calculating the correlation of anomalous differences in the two parts, has been widely used to evaluate the utility of anomalous differences in SAD experiments.

#### 1.2.3. Metrics requiring known structure   

Even in the absence of measurement errors, the anomalous substructure does not fully account for the observed anomalous differences, since this also contains a contribution from atoms that are not detectable from the signal owing to their individual contributions being very weak (for example having a low anomalous scattering factor, low occupancy or high atomic displacement parameters) or from anomalously scattering atoms in the solvent continuum (Fourme *et al.*, 1995 ▸). However, the cumulative effect of such atoms can be substantial in experiments such as sulfur SAD carried out at longer wavelengths (for example 1.8 Å), where the anomalous signal from C, N and O atoms becomes significant (Hendrickson, 2014 ▸), or in SAD experiments with heavy-atom soaks, where there are many minor sites, and even in selenomethionine SAD experiments, where the selenomethionine side chains might have multiple conformations.

In this work, we will define the ‘useful anomalous correlation’ as the correlation between measured anomalous differences and the ideal anomalous differences that correspond to the final refined structure considering anomalous scattering from the detectable anomalous substructure in the crystal only. This correlation of course cannot be calculated directly unless the structure has been solved. Nevertheless, we will show here that it is a useful way of quantifying the information that is present in individual anomalous differences. The useful anomalous correlation can be thought of as describing the fraction of the measured anomalous differences that correspond to the ideal anomalous differences coming just from the substructure. It is also affected (typically decreased) by measurement errors or radiation damage. As the useful anomalous correlation reflects the information available for phase calculation, it is anticipated to be related to the quality of the electron-density map that can be obtained from a SAD experiment (*cf.* Zwart, 2005 ▸, where the correlation of the anomalous differences to the values of the true anomalous structure factor *F*_A_ is related to the ease of SAD structure determination).

A second important metric is the ‘anomalous signal’, defined here, as in the work of others, as the peak height in an anomalous difference Fourier at the coordinates of the atoms in the anomalous substructure (Yang *et al.*, 2003 ▸). The anomalous signal can also only be calculated after the structure has been solved. We will show here that it is a good measure of the total information present per site in the substructure , Jane S. Richardsonin all the anomalous differences in a data set. It can be calculated from the observed anomalous differences and an atomic model for the structure (without the need to consider anomalous scattering from the structure or substructure). The anomalous signal has been found to be closely related to whether the anomalous substructure can be determined (Yang *et al.*, 2003 ▸; Liu *et al.*, 2011 ▸, 2013 ▸; Akey *et al.*, 2014 ▸; Bunkóczi *et al.*, 2014 ▸; Weinert *et al.*, 2015 ▸).

### 1.3. Objectives   

In this work, we develop a simple framework for describing the relationships between measured anomalous differences, useful anomalous correlation and the anomalous signal. We show that the anomalous signal can be used to estimate the probability of determining the anomalous substructure and that the useful anomalous correlation can be used to estimate the expected quality of the electron-density map that can be calculated once the substructure is known.

## 2. Methods   

### 2.1. Structure-factor relationships and anomalous differences in anomalous scattering   

**Figure 1:**
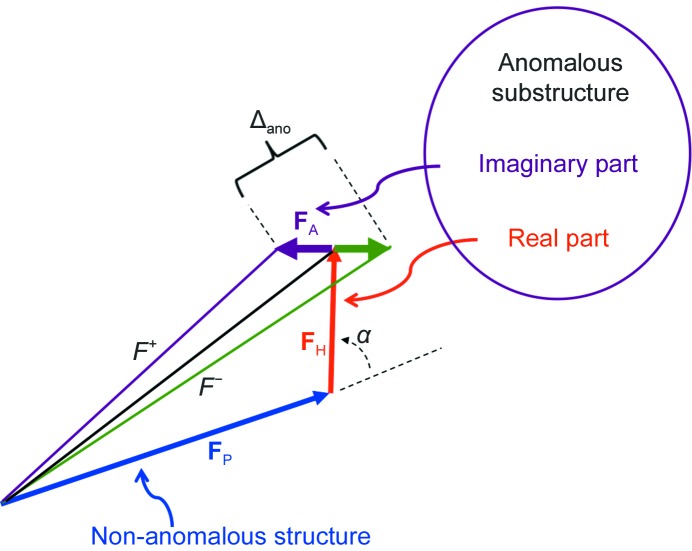
Diagram of the relationships between structure factors and anomalous differences. Structure factors corresponding to an acentric reflection with indices (*h*, *k*, *l*) and to its Bijvoet mate with indices (−*h*, −*k*, −*l*) are given. The structure factors for the Bijvoet mate are reflected across the *x* axis for clarity in presentation. The structure factor for the non-anomalous atoms in the structure is designated as **F**_P_. The part of the structure factor originating from the real part of the form factor for the atoms in the anomalous substructure (*f* ^o^ + *f*′) is shown as **F**_H_ and the part of this structure factor coming from the imaginary part of the form factor (*if*′′) is shown as **F**_A_.

We represent the scattering factor (form factor) for the anomalously scattering atoms in the structure as
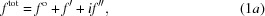
where *f* ^o^ + *f*′ is the real part of the form factor and *if*′′ is the imaginary part. Fig. 1 ▸ illustrates the structure-factor relationships contributing to anomalous scattering for a particular reflection in the simple case where there is a single type of anomalous scatterer in the structure (for example sulfur or selenium). The structure factor for all of the nonscattering or weakly anomalously scattering atoms (carbon, nitrogen, oxygen *etc.*) is written here as **F**_P_. The structure factor arising from the real part of the scattering factor *f* ^o^ + *f*′ for the anomalously scattering atoms is **F**_H_, and the part arising from the imaginary part of the scattering factor *if*′′ for these atoms is **F**_A_. The structure factors (**F** ^+^, **F** ^−^) for this Bijvoet pair of reflections are then given (after reflection of the **F** ^−^ member across the *x* axis; *c.f.* Kartha & Parthasarathy, 1965 ▸; Dauter *et al.*, 2002 ▸) by
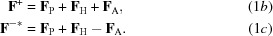
We will assume here that the structure factor arising from the imaginary part of the scattering factor for these atoms (**F**_A_) is small relative to the other components. In this case, we can write an approximation for the difference in magnitudes between the members of this Bijvoet pair of reflections (*F* ^+^ − *F* ^−^). This ‘anomalous difference’ (Δ_ano_) is approximately given by
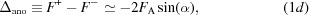
where α is the angle between the structure factors **F**_P_ and **F**_H_ (Fig. 1 ▸).

### 2.2. Contributions to the anomalous signal   

The anomalous signal (*S*_ano_; Yang *et al.*, 2003 ▸) is the mean peak height in an anomalous difference Fourier map at the coordinates of the anomalously scattering atoms, normalized to the r.m.s. value of the anomalous difference Fourier map. The anomalous difference Fourier map is calculated with coefficients based on the anomalous differences in (1*d* [Disp-formula fd3]),
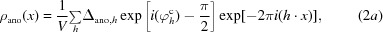
where Δ_ano,*h*_ is the anomalous difference for this reflection for reflection *h* and φ*_h_* ^c^ is the phase of the structure factor for the non-anomalous part of the structure (*F*_P_). The anomalous signal *S*_ano_ is then
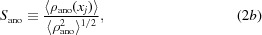
where ρ_ano_(*x_j_*) is the value of the anomalous difference Fourier at the position of the *j*th anomalously scattering atom and 〈ρ^2^_ano_〉^1/2^ is the r.m.s. of the map.

An anomalous difference Fourier is typically calculated in order to show the positions of the atoms in the anomalous substructure. It is related to the ideal Fourier map for these atoms, except that the coefficients *F*_H,*h*_exp[*i*(φ*_h_* ^c^ + α_*h*_)] in the ideal Fourier map are replaced by Δ_ano,*h*_exp[*i*(φ*_h_* ^c^ − π/2)] in the anomalous difference Fourier. We can see that this is reasonable by rearranging these coefficients slightly and by considering (1*d*). The coefficients in the ideal Fourier map are
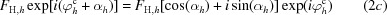
and the coefficients in the anomalous difference Fourier map (using 1*d*) are
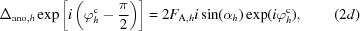
where *h* = (*h*, *k*, *l*) are the indices of a reflection, Δ_ano,*h*_ is the anomalous difference for this reflection, φ*_h_* ^c^ is the phase of the structure factor for the non-anomalous part of the structure (*F*_P_) calculated from the known structure, α_*h*_ is the angle between the structure factor arising from the real part of the form factor for the anomalous substructure (**F**_H_) and the structure factor for the non-anomalous part of the structure factors (**F**_P_; *cf*. Fig. 1 ▸). Inspecting (2*c*) and (2*d*), it can be seen that the anomalous difference Fourier represents just the sine term in (2*c*), and with a factor of 2*F*_A,*h*_ (twice the anomalous structure-factor amplitude) instead of *F*_H,*h*_ (the real structure-factor amplitude). It is therefore reasonable to expect that this map will have peaks at the positions of atoms in the anomalous substructure, but that the map will have a high level of intrinsic noise owing to missing the cosine term from (2*c*).

#### 2.2.1. Anomalous signal for an ideal anomalous difference Fourier   

We can now calculate how high the anomalous signal would be expected to be for an ideal anomalous difference Fourier (*i.e.* one with no measurement errors). Assuming that there is only one type of anomalous scatterer present, the structure factor **F**_A_ is perpendicular to **F**_H_ (Fig. 1 ▸) and their magnitudes are related for a particular reflection *h* by a factor *a* that corresponds to the ratio of anomalous to real scattering for the anomalous substructure and that depends on the resolution of the reflection,
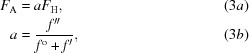
where *f* ^o^ + *f*′ and *f*′′ are the real and imaginary parts of the scattering factor for the atoms in the anomalous substructure at the resolution of reflection *h* (1*a*). As the imaginary part of the scattering is normally owing to core electrons close to the nucleus, its real-space image can be adequately represented by a delta function whose Fourier transform is constant as a function of resolution. Consequently, the values of *f*′′ and *f*′ are typically assumed to be constant with resolution but the value of the form factor *f* ^o^ falls off with resolution (Chantler, 1995 ▸; Hendrickson, 2014 ▸).

We can use these relationships and (2*c*) and (2*d*) to calculate the expected value of an anomalous difference Fourier map at the coordinates of the atoms in the substructure as well as the expected r.m.s. value of the map. Without loss of generality, in this analysis we will assume that the space-group setting chosen is a primitive setting (without centering). The value of the density in an anomalous difference Fourier map at coordinates represented by *x* = (*x*, *y*, *z*) can be written using (1*d*) and (2*b*) as
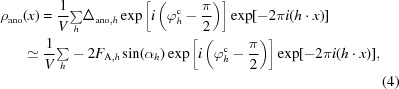
where *h*, Δ_ano,*h*_, φ_*h*_^c^ and α_*h*_ are as in (2*c*) and (2*d*) and *V* is the volume of the unit cell. After rearrangement and using the scale factor *a* given above between the anomalous structure-factor amplitude (*F*_A_) and the real part of the structure-factor amplitude for the substructure (*F*_H_), this expression for the density in an anomalous difference Fourier becomes
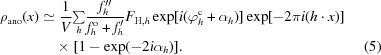



The structure factor arising from the real part of the form factor for the anomalous substructure (**F**_H,*h*_; Fig. 1 ▸) can be calculated from the scattering factors (*f_h_* ^o^ + *f*_*h*_′), coordinates (*x_j_*) and atomic displacement factors (*B_j_*) of the substructure as
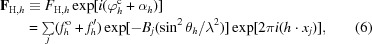
where sin(θ_*h*_) is the scattering angle for reflection *h* and λ is the wavelength of the X-rays used in the experiment. Substituting this expression for **F**_H,*h*_ into the approximation for the density in an anomalous difference Fourier gives
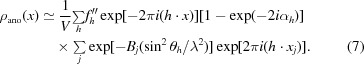



If all the atoms in the anomalous substructure are assumed to have identical atomic displacement factors (*B_j_* = *B*), then this can be simplified slightly and rearranged to read
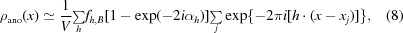
where the factors *f*_*h*,*B*_ are the anomalous scattering factors adjusted for the effects of the atomic displacement factor *B* at the resolution of reflection *h* and are given by
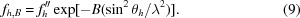



Noting that the expected value of the sum over all reflections 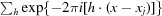 is zero unless *x* is approximately equal to one of the coordinates *x_j_* of an atom in the anomalous substructure, and noting that the phase angle α_*h*_ is the phase difference between the structure factor for the non-anomalous part of the structure and the anomalous substructure and is therefore essentially independent of the anomalous substructure, the expected value of the map 〈ρ_ano_(*x_j_*)〉 at atomic positions corresponding to the substructure is given by
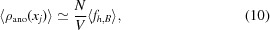
where *N* is the number of reflections (including the entire sphere, not just the asymmetric unit of the structure factors) used to calculate the map, *V* is the volume of the unit cell and 〈*f*_*h*,*B*_〉 is the mean value of the anomalous scattering factors (9) over all of the reflections included in the analysis.

A similar calculation can be made to estimate the mean-square value of the anomalous difference Fourier map and from it the r.m.s. of the map. The mean-square value of the density in this map is given by
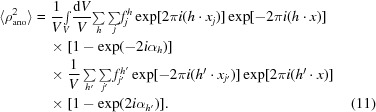
The expected value of the mean-square density in the anomalous difference Fourier map is then given by
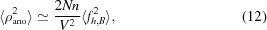
where, as before, *N* is the number of reflections and *V* is the volume of the unit cell. The number of atoms in the anomalous substructure in the entire unit cell is *n*, and 〈*f* ^2^_*h*,*B*_〉 is the mean-square value of the imaginary component of the anomalous scattering factors, including atomic displacement factors (9), over all of the reflections included in the analysis.

The anomalous signal is the ratio of the expected value of the density at coordinates of atoms in the anomalous substructure to the r.m.s. value of the map for the model-phased anomalous difference Fourier map. This can now be estimated,
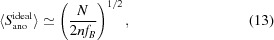
where *N* and *n* refer to the number of reflections including the entire sphere and the number of sites in the entire unit cell, respectively, and where the factor *f_B_* is the second moment of the values of the scattering factors,
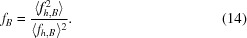
Writing the total number of reflections as the number of symmetry operators *N*_sym_ times the number of unique acentric reflections *N*_refl_ times two (for Bijvoet pairs of reflections),
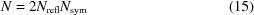
and writing the number of atoms in the anomalous substructure for the entire crystal as the number of atoms in the substructure in the asymmetric unit times the number of symmetry operators, 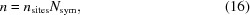
we can rewrite the expected anomalous signal in an ideal anomalous difference Fourier map as
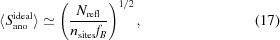
where *N*_refl_ is the number of unique noncentrosymmetric reflections and *n*_sites_ is the number of unique sites in the substructure. As noted at the beginning of the section, this analysis assumes that the space-group setting chosen is chosen to be primitive. As the number of unique reflections and the number of unique sites do not depend on the setting chosen, (17) can be applied equally well to centered space groups. Sites that are at special positions do require special treatment in (16), however (a site on a twofold will count as half the amount of a site on a general position).

#### 2.2.2. Anomalous signal for a realistic anomalous difference Fourier   

In this section, we will expand the calculation of expected anomalous signal to realistic cases where errors can be present in the measurement and where there may be significant anomalous scattering from minor sites. We can write a simple expression for contributions to an observed anomalous difference (Δ_ano_^obs^),
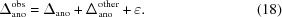
Here, Δ_ano_ is the ‘useful’ anomalous difference owing to the anomalous substructure, Δ_ano_^other^ is the (true, but not useful) anomalous difference owing to all other anomalously scattering atoms and minor sites for the anomalous substructure and ∊ is the error in measurement (including the effects of radiation damage). From this expression it can be seen that the anomalous contributions (Δ_ano_^obs^) from sites that are not part of the anomalous substructure and errors in measurement have similar overall effects on the observed anomalous differences.

We will assume that the observed anomalous difference and each term contributing to it have expected values of zero. Further, we will assume that each term contributing to the anomalous difference is uncorrelated with the other terms. We define a normalized variance, *E* ^2^, as the ratio of the sum of the variances of the anomalous contribution from sites not part of the substructure Δ_ano_^other^ and the errors in measurement ∊ to the mean-square value of the useful anomalous difference Δ_ano_,
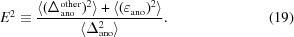
With this definition, we are in a position to calculate the expected value of the anomalous signal in the case where minor sites and measurement errors are present. The density in such a realistic anomalous difference Fourier map can be written (see equation 4) as
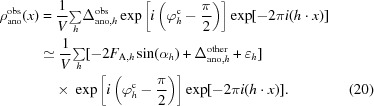
As in the previous section, this can be simplified and rearranged, yielding an expression for the density in an anomalous difference Fourier that is the sum of the density for a perfect anomalous difference Fourier and a term that contains the contributions from minor sites and errors in measurement,
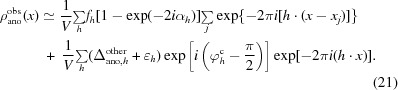
As the expected value of each of the two error terms is zero, the expected value of this density at the coordinates of atoms in the anomalous substructure is the same as in the case without errors (8), with the expected value given in (10). The expected value of the mean-square density in this map, however, is now higher owing to the error terms. It is given (compare with equation 12) by
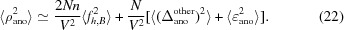
We can simplify this in several steps. From (1*d* [Disp-formula fd3]), it may be seen that the expected mean-square value of the anomalous difference corresponding to the substructure atoms (〈Δ^2^_ano_〉) is given by
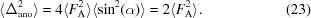
The value of 〈*F*_A_^2^〉 on the right-hand side of (23) can in turn be calculated in two steps. Firstly, substituting (3*a*) and (3*b*) into (6), assuming again that the atoms in the anomalous sub­structure are assumed to have identical atomic displacement factors (*B_j_* = *B*), and then substituting in (9) yields an expression relating the magnitude *F*_A_ to the anomalous scattering factor *f*_*h*,*B*_ from (9) and the coordinates of the anomalously scattering atoms *x_j_*,
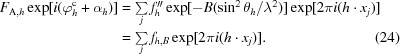
The mean-square value 〈*F*_A_^2^〉 can then be calculated as
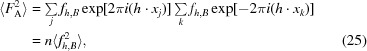
where *n* is the number of sites in the asymmetric unit of the crystal. Then, substituting (25) into (23), we have
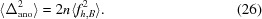
Finally, using (26) along with the definition of *E* ^2^ in (19) and rearranging, we can simplify the estimate of the mean-square density in the anomalous difference Fourier map with errors in (22) to read
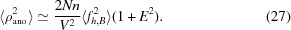
Comparing (12) and (27), we see that the mean-square value of the density overall in the anomalous difference Fourier map is larger than that in a perfect anomalous difference Fourier map by the factor 1 + *E* ^2^. As noted above (see equations 8, 10 and 21), the mean value of the density in the anomalous difference Fourier map with errors at coordinates of atoms in the anomalous substructure is the same as that in a perfect map. This leads to an expression for the anomalous signal in the presence of errors in measurement and minor sites,
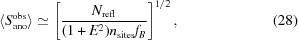
where the second moment of the values of the scattering factors *f* *_B_* is given in (14). We can relate the error term 1 + *E* ^2^ to the correlation (CC_ano_) between the measured anomalous differences and the anomalous differences owing to the sub­structure atoms. The useful anomalous correlation CC_ano_ is given by
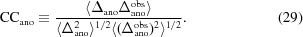
Using (18) and (19), it may be shown that the expected value of the useful anomalous correlation is given by
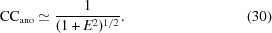
This yields a simple formula for the expected anomalous signal in the presence of minor sites and errors in measurement,
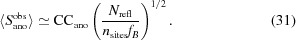
Comparison with (17) shows that the expected anomalous signal in a realistic case is simply equal to its expected value in an ideal case multiplied by the useful anomalous correlation CC_ano_.

### 2.3. Test data from the PDB   

We downloaded data sets from the PDB to serve as test cases for our analyses. The data consisted of 218 MAD and SAD data sets from 113 different PDB entries with diffraction data extending to resolutions from 1.2 to 4.5 Å. The MAD PDB entries were split into individual data sets for each wavelength of X-rays used to measure diffraction data. The *PHENIX* (Adams *et al.*, 2010 ▸) tool *phenix.sad_data_from_pdb* was used to extract the individual data sets from PDB entries. The PDB entries used were 1vjn, 1vjr, 1vjz, 1vk4, 1vkm (Levin *et al.*, 2005 ▸), 1vlm, 1vqr (Xu *et al.*, 2006 ▸), 1xri (Aceti *et al.*, 2008 ▸), 1y7e, 1z82, 1zy9, 1zyb, 2a2o, 2a3n, 2a6b, 2aj2, 2aml, 2avn, 2b8m, 2etd, 2etj, 2ets (Kozbial *et al.*, 2008 ▸), 2etv, 2evr (Xu, Sudek *et al.*, 2009 ▸), 2f4p, 2fea (Xu *et al.*, 2007 ▸), 2ffj, 2fg0 (Xu, Sudek *et al.*, 2009 ▸), 2fg9, 2fna (Xu, Rife *et al.*, 2009 ▸), 2fqp, 2fur, 2fzt, 2g0t, 2g42, 2gc9, 2nlv (Hwang *et al.*, 2014 ▸), 2nuj, 2nwv, 2o08, 2o1q, 2o2x, 2o2z, 2o3l, 2o62, 2o7t, 2o8q, 2obp, 2oc5, 2od5, 2od6, 2oh3, 2okc, 2okf (Hwang *et al.*, 2014 ▸), 2ooj, 2opk, 2osd, 2otm, 2ozg, 2ozj, 2p10, 2p4o, 2p7h, 2p7i, 2p97, 2pg3, 2pg4, 2pgc, 2pim, 2pn1, 2pnk, 2ppv, 2pr1, 2pr7, 2prv, 2pv4, 2pw4, 2wcd (Mueller *et al.*, 2009 ▸), 2xdd (Fineran *et al.*, 2009 ▸), 2zxh (Osawa *et al.*, 2009 ▸), 3caz, 3din (Zimmer *et al.*, 2008 ▸), 3dto, 3fx0 (Lo *et al.*, 2009 ▸), 3guw, 3gw7, 3hxk, 3hxp, 3lml, 3mv3 (Hsia & Hoelz, 2010 ▸), 3ov0 (Pokkuluri *et al.*, 2011 ▸), 3pg5, 3zgx (Bürmann *et al.*, 2013 ▸), 3zxu (Schmitzberger & Harrison, 2012 ▸), 4acb (Leibundgut *et al.*, 2004 ▸), 4asn (Aylett & Lowe, 2012 ▸), 4bql (Lindås *et al.*, 2014 ▸), 4cb0 (Mechaly *et al.*, 2014 ▸), 4cbv (Boudes *et al.*, 2014 ▸), 4fsx (Du *et al.*, 2012 ▸), 4g9i (Tominaga *et al.*, 2012 ▸), 4gkw (Qiao *et al.*, 2012 ▸), 4h6y (He *et al.*, 2013 ▸), 4hkr (Hou *et al.*, 2012 ▸), 4hnd (Zhou *et al.*, 2014 ▸), 4ifq (Sampathkumar *et al.*, 2013 ▸), 4lck (Zhang & Ferré-D’Amaré, 2013 ▸), 4nsc (Wang *et al.*, 2014 ▸), 4nt5 (Zhou & Springer, 2014 ▸), 4px7 (Fan *et al.*, 2014 ▸), 4q8j (Schäfer *et al.*, 2014 ▸), 4qka (Gao & Serganov, 2014 ▸) and 4tq5 (Huang *et al.*, 2014 ▸).

### 2.4. Software availability   

We have developed software as part of the *PHENIX* software suite (Adams *et al.*, 2010 ▸) that calculates the anomalous signal and anomalous correlation in a data set (*phenix.anomalous_signal*).

## 3. Results and discussion   

### 3.1. Dependence of the anomalous signal *S*_ano_^obs^ on CC_ano_, *N*_refl_, *n*_sites_ and *f_B_*   

The key theoretical result from this work (equation 31) is that the expected anomalous signal *S*_ano_^obs^ for a SAD data set is related in a very simple way to the useful anomalous correlation CC_ano_, the number of unique reflections *N*_refl_, the number of sites in the anomalous substructure *n*_sites_ and the second moment of the scattering factors for the anomalous substructure *f_B_*,
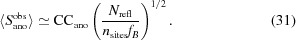
In (31) the anomalous signal *S*_ano_^obs^ is the mean value of a model-phased anomalous difference Fourier at the coordinates of atoms in the anomalous substructure. The anomalous signal is the principal metric in this work for the useful signal in a SAD data set. The useful anomalous correlation CC_ano_ is the correlation between measured anomalous differences and those expected of an ideal structure where the only anomalous scatterers are those in the anomalous substructure and where there are no errors in measurement (29). The useful anomalous correlation CC_ano_ is the principal metric in this work for similarity between measured and ideal anomalous differences. The number of reflections *N*_refl_ includes all acentric reflections that are unique under space-group symmetry, and the number of sites *n*_sites_ is the number contained in the asymmetric unit of the crystal. The factor *f_B_* (14) is the second moment of the scattering factors corresponding to the anomalous substructure. It is related to the fall-off with resolution of the scattering from the anomalous substructure because it includes the atomic displacement factors (assumed to all be equal; *B* *_j_* = *B*; equation 9).

#### 3.1.1. Anomalous signal for an idealized crystal   

The significance of (31) is that it shows how the anomalous signal depends on CC_ano_, *N*_refl_, *n*_sites_ and *f_B_*. If the data were measured perfectly and if there were no anomalous scatterers other than those in the substructure, then the useful anomalous correlation CC_ano_ would be unity. Further, if all of the anomalous scatterers had atomic displacement factors of zero (*B* = 0), then the second moment of the scattering factors *f_B_* would also be unity. The expected value 〈*S*_ano_^*B*=0^〉 of the anomalous signal in this ideal case with atomic displacement factors of zero is then simply equal to the square root of the ratio of unique reflections to unique sites,
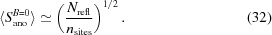
(32) indicates that anomalous signal increases with the number of unique reflections and decreases with the number of unique sites in the anomalous substructure in a simple way. In particular, if there are more sites in the substructure then correspondingly more reflections must be measured to obtain the same anomalous signal.

In this ideal case with atomic displacement factors of zero, the maximum possible expected anomalous signal 〈*S*_ano_^max^〉 for a crystal is obtained if there is a single site in the anomalous substructure (32). The value of the maximum possible expected anomalous signal is just the square root of the number of reflections,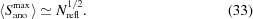.

#### 3.1.2. Dependence of the anomalous signal for an idealized crystal on resolution   

Up to this point, the influence of the resolution of the data on the anomalous signal has not been considered. If the anomalous scatterers in an otherwise idealized crystal have nonzero atomic displacement factors, then the contributions from these anomalous scatterers will be resolution-dependent (simply owing to the fall-off of intensities with resolution). Correspondingly, the anomalous signal (the peak heights at coordinates of anomalously scattering atoms in the anomalous difference Fourier) will not increase as the square root of the number of reflections as in (33), but rather by some smaller factor. This relationship is given in (17), where the effect of the fall-off of intensities with resolution is captured by the factor *f_B_*, the value of the second moment of the scattering factors including atomic displacement factors. If the anomalously scattering atoms have nonzero atomic displacement factors, the value of the second moment of the scattering factors, *f_B_* (14), will be greater than one. In such an idealized case, the anomalous signal 〈*S*_ano_^ideal^〉 will be depend on the resolution of the data through the factor *f_B_* in (17),
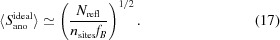
The second moment of the scattering factors *f_B_* depends on how much the scattering factors vary over the resolution of the measured reflections (*cf.* equation 14, noting that a distribution with many large and many small values will have a large value of the second moment while a narrow distribution will have a small second moment).

#### 3.1.3. Anomalous signal for anomalous data measured with errors from a real crystal   

For a real crystal, some of the true anomalous differences will come from minor sites of the principal anomalously scattering atom and from the weak anomalous scattering from atoms not considered to be part of the anomalous substructure. Furthermore, the anomalous differences will be measured with experimental errors. In this case the measured anomalous differences will have a correlation CC_ano_ to those measured perfectly from an ideal crystal. The consequence of a useful anomalous correlation of less than unity is that the anomalous signal will be reduced based on this correlation,
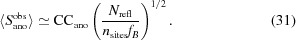
In this realistic case, the overall expected anomalous signal is just the product of the useful anomalous correlation and the square root of the number of reflections (*N*_refl_) divided by the number of sites in the anomalous substructure and the second moment *f_B_* of the scattering factors for the anomalous sub­structure.

#### 3.1.4. Anomalous signal and resolution of the data included in the calculation   

In this analysis (31), the anomalous correlation CC_ano_ is the overall average for the entire data set. As the accuracies of anomalous differences typically decrease at high resolution, if additional high-resolution, low-accuracy anomalous differences are included in a data set, then the overall value of CC_ano_ will decrease. Inspecting (31), it can be seen that as the resolution of data included in the calculation increases, the net effect on the anomalous signal is a combination of three factors. These are (i) an increase owing to the number of reflections *N*_refl_, (ii) a decrease owing to the decrease in average anomalous correlation CC_ano_ and (iii) a decrease owing to the increase in the second moment *f_B_* of the scattering factors for the anomalous substructure, averaged over all reflections included. The combined effects of increasing the number of reflections and increasing the average second moment of the scattering factors for the anomalous substructure, as discussed above, is that adding reflections that have negligible contributions from the anomalous substructure (*e.g.* at resolutions where the structure factors are essentially zero) has no effect on the overall anomalous signal.

The net effect of increasing the number of reflections and decreasing the anomalous correlation, however, can be either an increase or a decrease in the anomalous signal. If high-resolution data were measured with accuracy similar to that of lower resolution data, then the anomalous correlation CC_ano_ would be approximately constant as additional data are included and the anomalous signal would increase as the square root of the number of reflections. In contrast, if high-resolution data were measured with poor accuracy and the anomalous differences at these resolutions were essentially random, then the additional data would contribute only noise to the anomalous difference Fourier map (2*b* [Disp-formula fd5]). This would neither increase nor decrease the average peak height at positions of anomalously scattering atoms (the numerator in equation 2*b* [Disp-formula fd5]), but it would increase the overall r.m.s. of the map (the denominator in equation 2*b* [Disp-formula fd5]). Therefore, as expected, the inclusion of essentially random anomalous differences will decrease the overall anomalous signal *S*_ano_. This behavior can also be seen if (29) and (31) are examined. The anomalous correlation is defined in (29),
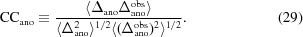
Imagine adding random anomalous differences, and suppose that they are about the same in magnitude as those that are already in the data set. In this case, the value of the r.m.s. observed anomalous difference 〈(Δ_ano_^obs^)^2^〉^1/2^ will not change. In contrast, the mean value of the product of the true and observed anomalous difference, 〈Δ_ano_Δ_ano_^obs^〉, will decrease. If *n* random anomalous differences are added to *N* well measured anomalous differences its value will decrease by a factor of *N*/(*N* + *n*). This means again that adding in anomalous differences that are random will reduce the overall anomalous signal *S*_ano_. We emphasize again that all of these effects are captured in (31), where the values of the anomalous correlation, number of reflections and second moment of scattering factors of the anomalously scattering atoms are all the overall values for all reflections considered.

#### 3.1.5. Comparison of expected and actual anomalous signal   

We carried out a comparison of the anomalous signal expected from (31) with the actual anomalous signal in data sets downloaded from the PDB. We used 113 MAD and SAD data sets from the PDB to serve as test cases for evaluating our theoretical analysis. The MAD data sets were split into separate ‘data sets’, each containing the data measured at one X-ray wavelength, yielding a total of 218 data sets for analysis. The high-resolution limits of these data sets ranged from 1.2 to 4.5 Å and the anomalously scattering atoms included selenium, sulfur, cobalt, mercury, zinc, nickel, iron, calcium, barium and iridium. Each data set was used to the full resolution limit except as noted below.

**Figure 2:**
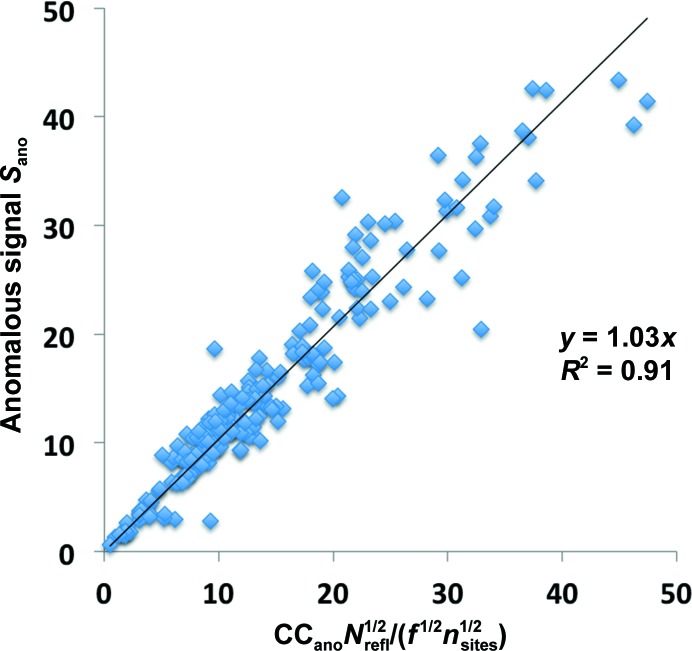
Comparison of anomalous signal estimated with (31) and the actual anomalous signal. Each point in the figure corresponds to data measured at one X-ray wavelength taken from one entry in the PDB. The *x* coordinate of each point is the anomalous signal estimated as described in the text using (31) and the *y* coordinate is the anomalous signal calculated directly from a model-phased anomalous difference Fourier map using (2*b*).

As all of the structures are known, we could calculate ideal anomalous differences Δ_ano_ based on the anomalous sub­structure in the model. We used these ideal differences along with the measured anomalous differences Δ_ano_^obs^ to calculate the useful anomalous correlation (CC_ano_; equation 17). From the X-ray data and model, we could identify the number of sites in the anomalous substructure (*n*_sites_) and the number of unique measured reflections (*N*_refl_). We calculated the second moment of the scattering factors of atoms in the substructure (*f_B_*) using (9) and (14) and using the mean value of the atomic displacement factors for the atoms in the substructure in (9). These calculations allowed us to estimate the value of the anomalous signal *S*_ano_^obs^ using (31). Note that all of the data are included in the calculation of the anomalous signal *S*_ano_^obs^. The resolution dependence of the anomalous signal is captured in the resolution dependence of the number of reflections, the anomalous correlation and the second moment of scattering factors of atoms in the substructure. We then obtained the actual value of the anomalous signal from the mean peak height at coordinates of atoms in the substructure in an anomalous difference Fourier map (2*b* [Disp-formula fd5]). Fig. 2 ▸ shows that the values of the anomalous signal obtained from (31) are very similar to the actual values obtained from an anomalous difference Fourier map using (2*b*), indicating that our simple theoretical analysis gives a good description of the overall contributions to the anomalous signal.

### 3.2. Relationship between the anomalous signal and the solution of the anomalous substructure   

**Figure 3:**
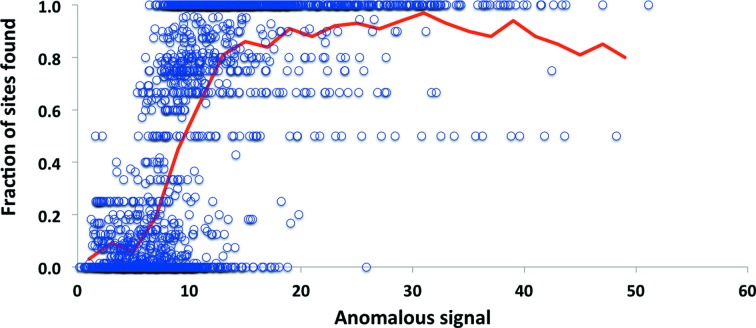
Success in substructure determination as a function of the anomalous signal in SAD data sets. Each point in the plot represents the fraction of the anomalous substructure found using likelihood-based methods for a complete or a resolution-truncated SAD data set as described in the text. The line represents the fraction of data sets where at least 50% of the sites in the substructure were found, as calculated in bins of resolution.

As noted above, the anomalous signal has been found to be a good indicator of whether the anomalous substructure can be obtained in a SAD experiment (Yang *et al.*, 2003 ▸; Liu *et al.*, 2011 ▸, 2013 ▸; Akey *et al.*, 2014 ▸; Bunkóczi *et al.*, 2014 ▸; Weinert *et al.*, 2015 ▸). Fig. 3 ▸ illustrates this relationship for 1874 complete and truncated data sets extracted from the 218 SAD data sets used above. These data sets were constructed by truncating the data at resolutions ranging from 1.5 to 6 Å. For each complete or truncated data set, the anomalous signal was calculated using an anomalous difference Fourier map and the coordinates of the atoms in the anomalous substructure. The same data were then used as input to the *HySS* substructure-search tool using likelihood-based substructure completion (Bunkóczi *et al.*, 2014 ▸), and the fraction of the true sites in the substructure is plotted in Fig. 3 ▸ as a function of the anomalous signal of the corresponding data set. Fig. 3 ▸ also illustrates the mean fraction of complete and truncated SAD data sets that are solved as a function of the anomalous signal. Those cases where at least 50% of the sites were found (sites placed within 3 Å of an atom or a symmetry-equivalent atom in the known substructure) were considered to be ‘solved’ for this analysis.

It can be seen from Fig. 3 ▸ that for data sets where the anomalous signal is less than about 7–10 few of the SAD data sets could be solved with likelihood-based methods, while for those where the anomalous signal is greater than about 10–15 most of the data sets could be solved, supporting the earlier observations that the anomalous signal is a good indicator of whether a SAD data set can be solved. The fraction of data sets that could be solved (the solid line in Fig. 3 ▸) reaches about 50% at an anomalous signal of about 9.

### 3.3. Relationship between useful anomalous correlation and the quality of phase calculation   

**Figure 4:**
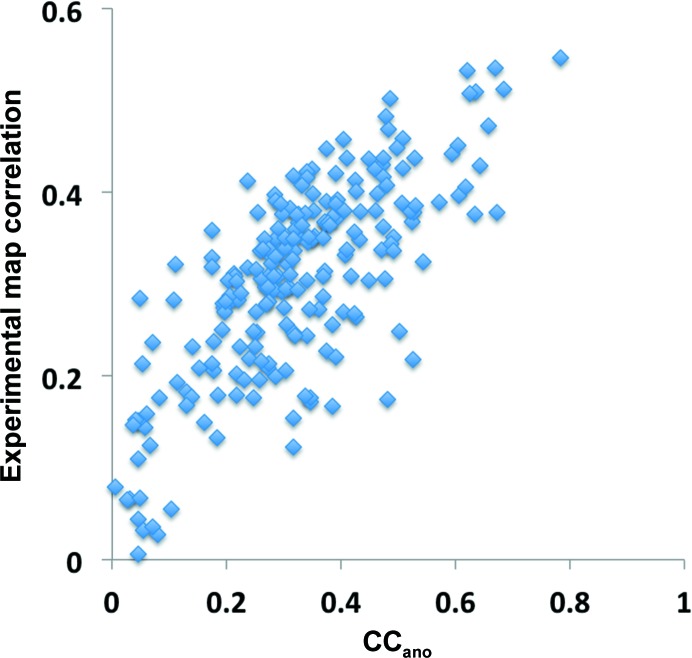
Phase accuracy as a function of useful anomalous correlation. Each point represents one SAD data set. The useful anomalous correlation is calculated from the correlation of model-based and measured anomalous differences. The measured anomalous differences are used along with the known anomalous substructure to calculate crystallographic phases. The phase accuracy is represented as the correlation between a map calculated using these phases and a map calculated using model phases.

The useful anomalous correlation (CC_ano_^obs^; equation 29) is a measure of how well the measured anomalous differences reflect those expected from the anomalous substructure in the crystal. As discussed by Zwart (2005 ▸), the accuracy of crystallographic phases that can be calculated from a set of anomalous differences using knowledge of the anomalous substructure is related to the useful anomalous correlation. Fig. 4 ▸ illustrates the relationship between useful anomalous correlation and the map correlation to a model-phased map obtained after phase calculations are carried out using *Phaser* (McCoy *et al.*, 2004 ▸) with the known anomalous substructure and the measured anomalous differences for the data sets shown in Fig. 2 ▸. Fig. 4 ▸ shows that for these data sets the map quality increases substantially with useful anomalous correlation.

### 3.4. Relationship between number of sites in the anomalous substructure and the anomalous signal   

(31) indicates that the anomalous signal is proportional to the inverse of the square root of the number of sites in the substructure. It is appropriate to consider how structure determination would be affected in a realistic situation by varying the number of sites. This is not quite as straight­forward as it might seem, as increasing the number of sites in the substructure would normally also increase the anomalous differences. Consequently, if all other crystal-size and data-collection conditions were the same, the crystal with more sites in the substructure would have a higher useful anomalous correlation (CC_ano_^obs^; equation 29), partially offsetting the decrease in anomalous signal expected from (31). Two limiting situations can be considered. In the case where anomalous differences are measured very accurately and the useful anomalous correlation is already very near unity, increasing the number of sites cannot increase the useful anomalous correlation (CC_ano_^obs^) further, so the increase in the number of sites will decrease the anomalous signal according to the square root of the number of sites as in (31).

In the more common case where the useful anomalous correlation (CC_ano_^obs^) is lower, increasing the number of sites will have a smaller effect on the anomalous signal than would be found with a constant value of the anomalous correlation. The extent of this effect can be seen by rewriting (31) to explicitly account for the contributions of the useful anomalous differences to the useful anomalous correlation by including the relationship between the normalized variance (*E* ^2^; equation 19) and the number of sites. Assuming independence of the terms in (18),[Disp-formula fd23] we can write an expression for the expected mean-square measured anomalous difference 〈(Δ_ano_^obs^)^2^〉, 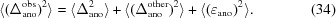
Then, considering (28), we can rewrite this in terms of the number of sites in the anomalous substructure *n*,
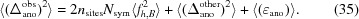
Substituting into (19), we obtain an expression for the normalized variance *E* ^2^ that depends on the number of sites, the mean-square anomalous difference Δ_ano_^other^ from sources other than the substructure, the errors in measurement ∊_ano_ and the scattering from an individual site in the substructure *f*_*h*,*B*_,
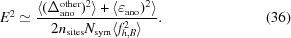
Factoring out the dependence of the normalized variance on the number of sites *n*_sites_, we can write that
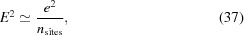
where *e* ^2^ is the ratio of the total mean-square errors in the anomalous differences to the expected mean-square useful anomalous differences for a single site in the substructure,
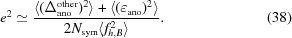
Using (37) and (38),[Disp-formula fd47] we can rewrite (31) (or equation 28) in a way that explicitly includes the number of sites and the ratio of the total mean-square errors in the anomalous differences to the expected mean-square useful anomalous differences for a single site in the substructure,
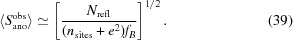
It can be seen from (39) that if the ratio *e* ^2^ is much smaller than the number of sites *n*_sites_ then increasing the number of sites will decrease the anomalous signal *S*_ano_^obs^ approximately according to the square root of the number of sites. This corresponds to the situation where the data are very accurately measured and there are only small anomalous differences arising from any atoms other than those in the substructure, as in §[Sec sec3.1.2]3.1.2. In contrast, if the ratio *e* ^2^ is comparable to or larger than the number of sites, then changing the number of sites will have a smaller effect, but the anomalous signal will still always decrease with increasing numbers of sites in the substructure. This corresponds to the situation in which the total mean-square error in the anomalous differences is comparable to or larger than the total mean-square useful anomalous difference.

## 4. Conclusions   

Our simple theory (31) shows how the anomalous signal in a SAD data set depends on the correlation of anomalous differences with those corresponding only to the anomalous substructure, the number of unique reflections measured, the number of sites in the substructure and the atomic displacement factors for the atoms in the substructure. Combining this with the empirical observation that the anomalous signal is a predictor of solving the anomalous substructure (Fig. 2 ▸) gives a clear idea of the features of the crystal and the experiment that determine whether the substructure can be obtained. (31) shows that even if the data are measured precisely, the anomalous signal is limited by several factors. These are the number of reflections measured, the number of sites in the substructure, the atomic displacement factors of the atoms in the substructure and the presence of minor sites. The limits on obtainable values of the anomalous signal can be used to ensure that substructure solution is at least possible when designing an experiment. (31) further shows that if errors in measurement are present, or if not all the anomalous scattering comes from the anomalous substructure, the anomalous signal is reduced by the value of the useful anomalous correlation CC_ano_^obs^ (the correlation of anomalous differences with those expected from a crystal where anomalous differences come only from the substructure and where there are no experimental errors). We emphasize that throughout this analysis the value of each parameter in (31) is the value corresponding to all reflections in the data set, and the parameters in (31) may change as higher resolution, lower accuracy data are included. Although including high-resolution, low-accuracy anomalous differences will increase the total number of reflections, which would seem to increase the anomalous signal through (31), this effect could be offset by the resulting lower value of the overall anomalous correlation CC_ano_^obs^. Consequently, in order to use (31) effectively to decide which data to include in an analysis it is important to consider how each parameter in (31) would be expected to change as additional data are included.

There are two principal bottlenecks in structure determination using the SAD method. One is finding the locations of atoms in the substructure, as discussed above, and the other is the calculation of crystallographic phases (Liu *et al.*, 2013 ▸). Fig. 4 ▸ indicates that the accuracy of experimental phases in the SAD method are closely related to the useful anomalous correlation CC_ano_^obs^. This is consistent with the observations of Zwart (2005 ▸) and provides a basis for predicting experimental map quality before and after the collection of SAD data.

Taken as a whole, our theoretical treatment of the contributions to the anomalous signal and useful anomalous correlation provide a foundation for evaluating whether a SAD experiment is likely to lead to successful substructure and phase determination.

## References

[bb1] Abrahams, J. P. & Leslie, A. G. W. (1996). *Acta Cryst.* D**52**, 30–42. 10.1107/S0907444995008754 15299723

[bb2] Aceti, D. J., Bitto, E., Yakunin, A. F., Proudfoot, M., Bingman, C. A., Frederick, R. O., Sreenath, H. K., Vojtik, F. C., Wrobel, R. L., Fox, B. G., Markley, J. L. & Phillips, G. N. Jr (2008). *Proteins*, **73**, 241–253. 10.1002/prot.22041 PMC4437517 18433060

[bb3] Adams, P. D. *et al.* (2010). *Acta Cryst.* D**66**, 213–221.

[bb4] Akey, D. L., Brown, W. C., Konwerski, J. R., Ogata, C. M. & Smith, J. L. (2014). *Acta Cryst.* D**70**, 2719–2729. 10.1107/S1399004714017556 PMC4188011 25286855

[bb5] Aylett, C. H. S. & Lowe, J. (2012). *Proc. Natl Acad. Sci. USA*, **109**, 16522–16527. 10.1073/pnas.1210899109 PMC3478639 23010931

[bb6] Berman, H. M., Westbrook, J., Feng, Z., Gilliland, G., Bhat, T. N., Weissig, H., Shindyalov, I. N. & Bourne, P. E. (2000). *Nucleic Acids Res.* **28**, 235–242. 10.1093/nar/28.1.235 PMC102472 10592235

[bb7] Bijvoet, J. M. (1954). *Nature (London)*, **173**, 888–891.

[bb8] Boudes, M., Sanchez, D., Graille, M., van Tilbeurgh, H., Durand, D. & Quevillon-Cheruel, S. (2014). *Nucleic Acids Res.* **42**, 5302–5313. 10.1093/nar/gku110 PMC4005675 24500202

[bb9] Buehner, M., Ford, G. C., Moras, D., Olsen, K. W. & Rossmann, M. G. (1974). *J. Mol. Biol.* **82**, 563–585. 10.1016/0022-2836(74)90249-6 4817797

[bb10] Bunkóczi, G., McCoy, A. J., Echols, N., Grosse-Kunstleve, R. W., Adams, P. D., Holton, J. M., Read, R. J. & Terwilliger, T. C. (2014). *Nature Methods*, **12**, 127–130. 10.1038/nmeth.3212 PMC4312553 25532136

[bb11] Bürmann, F., Shin, H., Basquin, J., Soh, Y., Giménez-Oya, V., Kim, Y., Oh, B. & Gruber, S. (2013). *Nature Struct. Mol. Biol.* **20**, 371–379. 10.1038/nsmb.2488 23353789

[bb12] Chantler, C. T. (1995). *J. Phys. Chem. Ref. Data*, **24**, 71.

[bb13] Colman, P. M., Jansonius, J. N. & Matthews, B. M. (1972). *J. Mol. Biol.* **70**, 701–724. 10.1016/0022-2836(72)90569-4 5083153

[bb14] Cowtan, K. (2006). *Acta Cryst.* D**62**, 1002–1011. 10.1107/S0907444906022116 16929101

[bb15] Cowtan, K. (2010). *Acta Cryst.* D**66**, 470–478. 10.1107/S090744490903947X PMC2852311 20383000

[bb16] Cowtan, K. D. & Main, P. (1996). *Acta Cryst.* D**52**, 43–48. 10.1107/S090744499500761X 15299724

[bb17] Dauter, Z. (2006). *Acta Cryst.* D**62**, 867–876. 10.1107/S0907444906023481 16855302

[bb18] Dauter, Z., Dauter, M. & Dodson, E. J. (2002). *Acta Cryst.* D**58**, 494–506. 10.1107/s090744490200118x 11856836

[bb19] Debreczeni, J. É., Bunkóczi, G., Ma, Q., Blaser, H. & Sheldrick, G. M. (2003). *Acta Cryst.* D**59**, 688–696. 10.1107/s0907444903002646 12657788

[bb20] Du, J., Zhong, X., Bernatavichute, Y. V., Stroud, H., Feng, S., Caro, E., Vashisht, A. A., Terragni, J., Chin, H. G., Tu, A., Hetzel, J., Wohlschlegel, J. A., Pradhan, S., Patel, D. J. & Jacobsen, S. E. (2012). *Cell*, **151**, 167–180. 10.1016/j.cell.2012.07.034 PMC3471781 23021223

[bb21] Emsley, P., Lohkamp, B., Scott, W. G. & Cowtan, K. (2010). *Acta Cryst.* D**66**, 486–501. 10.1107/S0907444910007493 PMC2852313 20383002

[bb22] Evans, P. (2006). *Acta Cryst.* D**62**, 72–82. 10.1107/S0907444905036693 16369096

[bb23] Fan, J., Jiang, D., Zhao, Y., Liu, J. & Zhang, X. C. (2014). *Proc. Natl Acad. Sci. USA*, **111**, 7636–7640. 10.1073/pnas.1403097111 PMC4040569 24821770

[bb24] Fineran, P. C., Blower, T. R., Foulds, I. J., Humphreys, D. P., Lilley, K. S. & Salmond, G. P. C. (2009). *Proc. Natl Acad. Sci. USA*, **106**, 894–899. 10.1073/pnas.0808832106 PMC2630095 19124776

[bb26] Fourme, R., Shepard, W., Kahn, R., l’Hermite, G. & Li de La Sierra, I. (1995). *J. Synchrotron Rad.* **2**, 36–48. 10.1107/S0909049594006680 16714785

[bb27] Fu, Z.-Q., Rose, J. P. & Wang, B.-C. (2004). *Acta Cryst.* D**60**, 499–506. 10.1107/S0907444904000617 14993675

[bb28] Furey, W. & Swaminathan, S. (1997). *Methods Enzymol.* **276**, 590–620. 10.1016/s0076-6879(97)77033-2 18488326

[bb29] Gao, A. & Serganov, A. (2014). *Nature Chem. Biol.* **10**, 787–792. 10.1038/nchembio.1607 PMC4294798 25086507

[bb30] Garman, E. (2003). *Curr. Opin. Struct. Biol.* **13**, 545–551. 10.1016/j.sbi.2003.09.013 14568608

[bb31] González, A. (2003). *Acta Cryst.* D**59**, 1935–1942. 10.1107/s0907444903017700 14573948

[bb32] Grosse-Kunstleve, R. W. & Adams, P. D. (2003). *Acta Cryst.* D**59**, 1966–1973. 10.1107/s0907444903018043 14573951

[bb33] He, X., Kuo, Y.-C., Rosche, T. J. & Zhang, X. (2013). *Structure*, **21**, 355–364. 10.1016/j.str.2013.01.001 PMC3595398 23375260

[bb34] Hendrickson, W. A. (2014). *Q. Rev. Biophys.* **47**, 49–93. 10.1017/S0033583514000018 PMC4128195 24726017

[bb35] Hendrickson, W. A. & Teeter, M. M. (1981). *Nature (London)*, **290**, 107–113. 10.1038/290107a0 PMC5536114 28769131

[bb36] Hou, X., Pedi, L., Diver, M. M. & Long, S. (2012). *Science*, **338**, 1308–1313. 10.1126/science.1228757 PMC3695727 23180775

[bb37] Howell, P. L. & Smith, G. D. (1992). *J. Appl. Cryst.* **25**, 81–86.

[bb38] Hsia, K.-C. & Hoelz, A. (2010). *Proc. Natl Acad. Sci. USA*, **107**, 11271–11276. 10.1073/pnas.1006297107 PMC2895078 20534429

[bb39] Huang, H., Levin, E. J., Liu, S., Bai, Y., Lockless, S. W. & Zhou, M. (2014). *PLoS Biol.* **12**, e1001911. 10.1371/journal.pbio.1001911 PMC4106721 25051182

[bb40] Hwang, W. C., Golden, J. W., Pascual, J., Xu, D., Cheltsov, A. & Godzik, A. (2014). *Proteins*, 10.1002/prot.24679.

[bb41] Jones, T. A., Zou, J.-Y., Cowan, S. W. & Kjeldgaard, M. (1991). *Acta Cryst.* A**47**, 110–119. 10.1107/s0108767390010224 2025413

[bb42] Kartha, G. & Parthasarathy, R. (1965). *Acta Cryst.* **18**, 745–749. 10.1107/s0365110x65001718 14289668

[bb43] Kozbial, P. *et al.* (2008). *Proteins*, **71**, 1589–1596. 10.1002/prot.22020 18324683

[bb44] Krojer, T., Pike, A. C. W. & von Delft, F. (2013). *Acta Cryst.* D**69**, 1303–1313. 10.1107/S0907444913013280 PMC3689534 23793157

[bb25] La Fortelle, E. de & Bricogne, G. (1997). *Methods Enzymol.* **276**, 472–494. 10.1016/S0076-6879(97)76073-7 27799110

[bb45] Langer, G., Cohen, S. X., Lamzin, V. S. & Perrakis, A. (2008). *Nature Protoc.* **3**, 1171–1179. 10.1038/nprot.2008.91 PMC2582149 18600222

[bb46] Leibundgut, M., Frick, C., Thanbichler, M., Böck, A. & Ban, N. (2004). *EMBO J.* **24**, 11–22. 10.1038/sj.emboj.7600505 PMC544917 15616587

[bb47] Levin, I. *et al.* (2005). *Proteins*, **59**, 864–868. 10.1002/prot.20420 15822122

[bb48] Lindås, A.-C., Chruszcz, M., Bernander, R. & Valegård, K. (2014). *Acta Cryst.* D**70**, 492–500. 10.1107/S1399004714000935 24531483

[bb50] Liu, Q., Liu, Q. & Hendrickson, W. A. (2013). *Acta Cryst.* D**69**, 1314–1332. 10.1107/S0907444913001479 PMC3689535 23793158

[bb49] Liu, Z.-J., Chen, L., Wu, D., Ding, W., Zhang, H., Zhou, W., Fu, Z.-Q. & Wang, B.-C. (2011). *Acta Cryst.* A**67**, 544–549. 10.1107/S0108767311037469 PMC3211246 22011470

[bb51] Lo, Y.-C., Lin, S.-C., Rospigliosi, C. C., Conze, D. B., Wu, C.-J., Ashwell, J. D., Eliezer, D. & Wu, H. (2009). *Mol. Cell*, **33**, 602–615. 10.1016/j.molcel.2009.01.012 PMC2749619 19185524

[bb52] McCoy, A. J., Storoni, L. C. & Read, R. J. (2004). *Acta Cryst.* D**60**, 1220–1228. 10.1107/S0907444904009990 15213383

[bb53] Mechaly, A. E., Sassoon, N., Betton, J.-M. & Alzari, P. M. (2014). *PLoS Biol.* **12**, e1001776. 10.1371/journal.pbio.1001776 PMC3904827 24492262

[bb54] Mueller, M., Grauschopf, U., Maier, T., Glockshuber, R. & Ban, N. (2009). *Nature (London)*, **459**, 726–730. 10.1038/nature08026 19421192

[bb55] North, A. C. T. (1965). *Acta Cryst.* **18**, 212–216.

[bb56] Osawa, T., Ito, K., Inanaga, H., Nureki, O., Tomita, K. & Numata, T. (2009). *Structure*, **17**, 713–724. 10.1016/j.str.2009.03.013 19446527

[bb57] Otwinowski, Z. (1991). *Proceedings of the CCP4 Study Weekend. Isomorphous Replacement and Anomalous Scattering*, edited by W. Wolf, P. R. Evans & A. G. W. Leslie, pp. 80–86. Warrington: Daresbury Laboratory.

[bb58] Otwinowski, Z. & Minor, W. (1997). *Methods Enzymol.* **276**, 307–326. 10.1016/S0076-6879(97)76066-X 27754618

[bb59] Pannu, N. S. & Read, R. J. (2004). *Acta Cryst.* D**60**, 22–27. 10.1107/s0907444903020808 14684888

[bb60] Parthasarathy, S. & Parthasarathi, V. (1974). *Acta Cryst.* A**30**, 649–654.

[bb61] Perrakis, A., Morris, R. & Lamzin, V. S. (1999). *Nature Struct. Biol.* **6**, 458–463. 10.1038/8263 10331874

[bb62] Pokkuluri, P. R., Londer, Y. Y., Duke, N. E., Pessanha, M., Yang, X., Orshonsky, V., Orshonsky, L., Erickson, J., Zagyanskiy, Y., Salgueiro, C. A. & Schiffer, M. (2011). *J. Struct. Biol.* **174**, 223–233. 10.1016/j.jsb.2010.11.022 21130881

[bb63] Qiao, R., Cabral, G., Lettman, M. M., Dammermann, A. & Dong, G. (2012). *EMBO J.* **31**, 4334–4347. 10.1038/emboj.2012.280 PMC3501224 23064147

[bb64] Sampathkumar, P. *et al.* (2013). *Structure*, **21**, 560–571. 10.1016/j.str.2013.02.005 PMC3755625 23499021

[bb65] Schäfer, I. B., Rode, M., Bonneau, F., Schüssler, S. & Conti, E. (2014). *Nature Struct. Mol. Biol.* **21**, 591–598. 10.1038/nsmb.2834 24880344

[bb66] Schmitzberger, F. & Harrison, S. C. (2012). *EMBO Rep.* **13**, 216–222. 10.1038/embor.2012.1 PMC3323139 22322944

[bb67] Schneider, T. R. & Sheldrick, G. M. (2002). *Acta Cryst.* D**58**, 1772–1779. 10.1107/s0907444902011678 12351820

[bb68] Shen, Q., Wang, J. & Ealick, S. E. (2003). *Acta Cryst.* A**59**, 371–373. 10.1107/s0108767303009115 12832817

[bb69] Strahs, G. & Kraut, J. (1968). *J. Mol. Biol.* **35**, 503–512. 10.1016/s0022-2836(68)80010-5 5673695

[bb70] Terwilliger, T. C. (2000). *Acta Cryst.* D**56**, 965–972. 10.1107/S0907444900005072 PMC2792768 10944333

[bb71] Terwilliger, T. C. & Berendzen, J. (1999). *Acta Cryst.* D**55**, 849–861. 10.1107/S0907444999000839 PMC2746121 10089316

[bb72] Terwilliger, T. C., Bunkóczi, G., Hung, L.-W., Zwart, P. H., Smith, J., Akey, D. & Adams, P. D. (2016). *Acta Cryst.* D**72**, 359–374. 10.1107/S2059798315019403 PMC4784667 26960123

[bb73] Terwilliger, T. C., Grosse-Kunstleve, R. W., Afonine, P. V., Moriarty, N. W., Zwart, P. H., Hung, L.-W., Read, R. J. & Adams, P. D. (2008). *Acta Cryst.* D**64**, 61–69. 10.1107/S090744490705024X PMC2394820 18094468

[bb74] Tominaga, T., Watanabe, S., Matsumi, R., Atomi, H., Imanaka, T. & Miki, K. (2012). *Acta Cryst.* F**68**, 1153–1157. 10.1107/S1744309112036421 PMC3497970 23027738

[bb75] Wang, B.-C. (1985). *Methods Enzymol.* **115**, 90–112. 10.1016/0076-6879(85)15009-3 4079800

[bb76] Wang, L., Yang, X., Li, S., Wang, Z., Liu, Y., Feng, J., Zhu, Y. & Shen, Y. (2014). *EMBO J.* **33**, 594–604. 10.1002/embj.201386523 PMC3989653 24514027

[bb77] Weeks, C. M., DeTitta, G. T., Miller, R. & Hauptman, H. A. (1993). *Acta Cryst.* D**49**, 179–181. 10.1107/S090744499200876X 15299558

[bb78] Weinert, T. *et al.* (2015). *Nature Methods*, **12**, 131–133. 10.1038/nmeth.3211 25506719

[bb81] Xu, Q. *et al.* (2006). *Proteins*, **62**, 292–296. 10.1002/prot.20611 16287129

[bb80] Xu, Q. *et al.* (2007). *Proteins*, **69**, 433–439. 10.1002/prot.21602 17654724

[bb79] Xu, Q., Rife, C. L. *et al.* (2009). *Proteins*, **74**, 1041–1049. 10.1002/prot.22325 PMC2692486 19089981

[bb82] Xu, Q., Sudek, S. *et al.* (2009). *Structure*, **17**, 303–313. 10.1016/j.str.2008.12.008 PMC2667786 19217401

[bb83] Yang, C., Pflugrath, J. W., Courville, D. A., Stence, C. N. & Ferrara, J. D. (2003). *Acta Cryst.* D**59**, 1943–1957. 10.1107/s0907444903018547 14573949

[bb84] Zhang, J. & Ferré-D’Amaré, A. R. (2013). *Nature (London)*, **500**, 363–366. 10.1038/nature12440 PMC3808885 23892783

[bb85] Zhou, Q., Li, J., Yu, H., Zhai, Y., Gao, Z., Liu, Y., Pang, X., Zhang, L., Schulten, K., Sun, F. & Chen, C. (2014). *Nature Commun.* **5**, 3552. 10.1038/ncomms4552 PMC3974213 24675427

[bb86] Zhou, Y. F. & Springer, T. A. (2014). *Blood*, **123**, 1785–1793. 10.1182/blood-2013-11-523639 PMC3962159 24394662

[bb87] Zimmer, J., Nam, Y. & Rapoport, T. A. (2008). *Nature (London)*, **455**, 936–943. 10.1038/nature07335 PMC7164768 18923516

[bb88] Zwart, P. H. (2005). *Acta Cryst.* D**61**, 1437–1448. 10.1107/S0907444905023589 16239720

